# Effects of Ball Milling Combined With Cellulase Treatment on Physicochemical Properties and *in vitro* Hypoglycemic Ability of Sea Buckthorn Seed Meal Insoluble Dietary Fiber

**DOI:** 10.3389/fnut.2021.820672

**Published:** 2022-01-28

**Authors:** Yulian Zhu, Xiaolong Ji, Michael Yuen, Tina Yuen, Hywel Yuen, Min Wang, Deandrae Smith, Qiang Peng

**Affiliations:** ^1^College of Food Science and Engineering, Northwest A&F University, Yangling, China; ^2^Henan Key Laboratory of Cold Chain Food Quality and Safety Control, Zhengzhou University of Light Industry, Zhengzhou, China; ^3^Puredia Limited, Xining, China; ^4^Department of Food Science and Technology, University of Nebraska, Lincoln, NE, United States

**Keywords:** hypoglycemic ability, insoluble dietary fiber, starch digestion inhibition, α-amylase activity inhibition, cellulase treatment

## Abstract

To improve the rough texture and hypoglycemic ability of sea buckthorn insoluble dietary fiber (IDF), a novel combined modification method was developed in this study. The IDF was treated with ball milling and cellulase treatment to obtain co-modified insoluble dietary fiber (CIDF). The physicochemical and functional properties of IDF, milled insoluble dietary fiber (MIDF), and CIDF were studied. After treatments, MIDF had smaller particle sizes and a looser structure, and CIDF exhibited a wrinkled surface and sparse porous structure according to scanning electron microscopy (SEM) and X-ray diffraction. Compared to IDF, MIDF and CIDF showed improved water-holding, oil-binding, and swelling capacities, improved by 16.13, 14.29, and 15.38%, and 38.5, 22.2, and 25.0%, for MIDF and CIDF, respectively. The cation exchange ability of modified samples showed improvement as well. Treatments also changed the fluidity of MIDF and CIDF. Due to the smaller particles and increased stacking, the bulk density (BD) and angle of repose of MIDF improved by 33.3% and 4.1° compared to IDF, whereas CIDF had a looser structure and thus decreased by 7.1% and 13.3° with increased fluidity. Moreover, the modification also enhanced the effects of CIDF on glucose adsorption, glucose diffusion inhibition, starch digestion inhibition, starch pasting interference, and α-amylase activity inhibition. In summary, IDF modified by ball milling combined with cellulose treatment could be developed as a functional ingredient for regulating glucose content.

## Introduction

Diabetes is a major public health problem and a substantial global economic burden (1). Relevant studies have shown that dietary fiber is an important regulator of the metabolic mechanism of blood glucose and lipid in the body. The addition of appropriate dietary fiber to the diet could effectively improve the symptoms of hyperglycemia and prevent type 2 diabetes (2). Researches have confirmed that dietary fiber can help to control postprandial blood glucose by slowing the absorption of carbohydrates and could also increase abdominal satiety to control the reduction of total calorie intake (3, 4). Insoluble dietary fibers (IDFs) could inhibit starch digestion through increasing molecule interactions and physical hindrance formation, thereby reducing the formation and utilization of glucose (3, 5). In addition, IDFs could also reduce the adsorption of glucose by inhibiting the diffusion of glucose in the intestine through the barrier effects of the particles, and also by directly adsorbing glucose (6, 7).

Sea buckthorn (*Hippophae rhamnoides*) belongs to the Elaeagnaceae family, and is widely cultivated in China, Central Asia, and Mongolia (3). Sea buckthorn seed meal is a by-product of the sea buckthorn processing industry and contains high levels of bioactive substances (8). Referring to the standard enzymatic gravimetric method of AOAC Official 991.43 (9), the recent study in our laboratory has shown that dietary fiber is the main component of sea buckthorn seed meal. The total dietary fiber content is 57.5/100 g, of which 98.8% is IDF and 1.2% is soluble dietary fiber. Related research on sea buckthorn seed meal IDF is only limited to simple extraction (10), and studies on its functional properties and modifications are also scarce.

In recent years, there has been an increasing interest in IDF from plant processing by-products due to its low cost, safety, and accessibility (11, 12). However, traditional extraction methods have limitations on the physicochemical and functional properties of IDFs. It has now been shown that physical, chemical, biological, and complex modification methods could be used to enhance the properties of IDF and improve its hypoglycemic ability (13). The main methods, currently used to modify IDFs, include ultrafine comminution (14), high-pressure homogenization (15), acid (16), and alkaline treatments (17), grafting with groups, such as hydroxyl and carboxyl groups (7), and enzymatic hydrolysis (18). IDF is composed of cellulose, hemicellulose, and lignin. The cellulose is surrounded by a tight spatial mesh of hemicellulose and lignin. Such physical shielding prevents cellulose from coming in contact with external substances and also prevents enzymatic degradation of the cellulose (19). To increase the specific surface area of the raw material, improve the accessibility of the enzyme to the substrate, and increase the efficiency of the enzymatic degradation of cellulose, it is necessary to choose the appropriate pretreatment of the material. Some pretreatments could break the link between lignin, cellulose, and hemicellulose, reducing the recalcitrance of the raw materials (19), whereas others can be effective in destroying the crystallinity of cellulose, increasing the porosity and specific surface area of the raw material, and improving the accessibility of cellulose to the enzymes (20). It was decided that mechanical comminution pretreatment combined with cellulase treatment could effectively improve the functional properties of IDF.

The objective of this study was to modify the IDF of sea buckthorn seed meal with ball milling and ball milling combined with cellulose treatment, characterize its structure and physicochemical features, and then evaluate its *in vitro* hypoglycemic effects. The current findings will provide a theoretical basis for the further utilization of sea buckthorn and develop the modified IDF as a functional ingredient.

## Materials and Methods

### Materials

Sea buckthorn seed meal was obtained from Puredia Limited (Xining, Qinghai, China). The cellulase (CEL-01 11,000 U/g) was obtained from Beijing Sunon Co. Ltd. The heat-stable α-amylase (A3176, 5 U/mg, Solid) was purchased from Sigma-Aldrich (St. Louis, MO, USA). The glucosidase was bought from Nanjing Duly Biotech Co., Ltd. (Nanjing, Jiangsu, China). Other chemicals used in this study were all of the analytical grade.

### Extraction of IDF

The IDF of sea buckthorn seed meal was prepared according to the previous method (21) with some modifications. Sea buckthorn seed meal was blended with distilled water (1:20 V/V) at the pH 9 and then incubated at 50°C for 60 min. After cooling to room temperature, the solution was centrifugated at 3,500 rpm for 10 min to collect the precipitate. This extraction process was performed two times. The precipitate was washed two times with distilled water (1:10 V/V) at 90°C and dried at 60°C and then passed through a 100-mesh sieve to obtain the IDF.

### Milling of IDF

The IDF was processed with a planetary ball mill (YXQM-2L, Mitr, Changsha, China) and mixed two times with the quantity of zirconia balls (3–5 mm diameter) in a 250-ml vessel at 600 rpm/min for 6 h. An intermittent method (run for 60 min and then cool it for 10 min) was carried out to maintain the milled insoluble dietary fiber (MIDF).

### Cellulase Treatment of MIDF

The cellulase treatment was based on the method of the previous study (22) with some modifications; 5 g of MIDF was mixed with 40 ml of 0.05 mol/L phosphate-buffered saline (PBS) buffer at the pH 5.5 with 0.04 g cellulase addition. The mixture was incubated at 70°C for 2 h with magnetic stirring. The reaction was terminated by putting the mixture into 90°C hot water for 15 min. The mixture was then cooled down and centrifugated at 4,000 rpm for 10 min to remove the supernatant. The co-modified insoluble dietary fiber (CIDF) was obtained by drying overnight at 60°C and passing through a 100-mesh sieve.

### Structural Characterization of Fibers

#### Scanning Electron Microscopy

A scanning electron microscopy (SEM) (S-3400N, Hitachi, Tokyo, Japan) was used to analyze the surface morphology of the samples. Referring to a previous study (23), 2 mg of sample was attached to a conductive adhesive and metalized with gold, and then, the images were collected at 400×, 3,000×, 6,000×, and 12,000×.

#### X-Ray Diffraction

The X-ray diffraction (XRD) patterns of samples were obtained from X-ray diffraction (D8 ADVANCE A25 Diffractometer, Bruker, Karlsruhe, Bundesrepublik Germany), at the operating voltage and current at 40 kV and 40 mA, scanning from 5 to 70° with the angle step of 0.02°.

#### Fourier Transfer-Infrared Spectrometry

The Fourier transfer-infrared spectrometry (FT-IR) spectrum of samples was detected using an FT-IR instrument (Vertex 70, Bruker, Germany) according to the previous study (24) with KBr disk as the background, scanning from 400 to 4,000 cm^−1^.

### Physicochemical Properties Determination

#### Water-Holding Capacity

Referring to the previous method (14), 1 g (M) of IDF sample was put in a tube (M_0_), mixed with 15 ml distilled water, and then incubated at room temperature for 2 h. After the treatment, the mixture was centrifugated at 4,000 rpm for 15 min. The supernatant was then removed. The wet weight with the centrifugal tube was recorded as M_1_. The supernatant was dried to a constant weight and recorded as M_2_. The waterholding capacity (WHC) was calculated by the following equation:

$$\begin{array}{l} \text{WHC}\left(\text{g}/\text{g}\right)=\left({\text{M}}_{1}-{\text{M}}_{2}-{\text{M}}_{0}\right)/\text{M} \end{array}$$

#### Oil-Binding Capacity

According to the previous study (25), 1 g (M) of IDF sample was put in a centrifuge tube (M_0_) and mixed with 25 ml corn oil, and then, the mixture was shaken in a 36°C water bath for 2 h. After the treatment, the oil was removed by centrifuging the mixture at 4,000 rpm for 30 min. The wet weight of the tube and sample was recorded as M_1_. The oil-binding capacity (OBC) was calculated by the following equation:

$$\begin{array}{l} \text{OBC}\left(\text{g}/\text{g}\right)=\left({\text{M}}_{1}-{\text{M}}_{0}-\text{M}\right)/\text{M} \end{array}$$

#### Swelling Capacity

Following the reported method (26), 1 g (M) of IDF sample was put in a volumetric cylinder and mixed with 10 ml distilled water. This volume was recorded as V_0_. The volume of the cylinder was recorded again as V_1_ after being kept for 24 h at room temperature. The swelling capacity (SC) was calculated by the following equation:

$$\begin{array}{l} \text{SC}\left(\text{ml}/\text{g}\right)=\left({\text{V}}_{0}-{\text{V}}_{1}\right)/\text{M} \end{array}$$

#### Bulk Density

The bulk density (BD) of samples was calculated according to the method reported in the previous study (27). A volumetric flask (2 ml) was weighed and recorded as W_1_, then filled with IDF, and then weighed and recorded again as W_2_. The BD was calculated by the following equation:

$$\begin{array}{l} \text{BD}\left(\text{g}/\text{ml}\right)=\frac{{\text{W}}_{2}\;-\;{\text{W}}_{1}}{2} \end{array}$$

#### Angle of Repose

Referred to the reported method (28), a funnel was fixed vertically 3 cm above a piece of paper. Then, 3 g of sample was poured into the funnel and allowed to flow through the spout and onto the paper. The flowing sample formed a cone on the paper, the radius and height of the corn were measured as R and H, the angle of repose (α) was calculated from the following equation:

$$\begin{array}{l} \text{α}=\text{arctan }\left(\frac{\text{H}}{\text{R}}\right) \end{array}$$

### Cation Exchange Capability

Based on the previous method (14) with some modifications, the acid sample was obtained by mixing 1 g IDF with 60 ml hydrochloric acid (0.1 M) for 24 h. The residue was then collected and washed extensively with distilled water until no Cl^−^ was detected, and then dried at 60°C. After that, 0.1 g of acidic sample was weighed and dispersed into 50 ml 5% NaCl solution, titrated with 0.01 M NaOH, and the pH of the mixture was recorded.

### Glucose Adsorption Ability

According to the previous method (29), 1 g of sample was mixed with 100 ml glucose solution at different concentrations (10, 50, 100, and 200 mmol/L) and incubated at 37°C for 6 h with shaking. After this, the mixture was centrifugated at 4,000 rpm for 20 min, the supernatant was collected, and the glucose content was measured using a biosensors analyzer (S-10, Sieman Technology Co., Ltd., Shenzhen, China).

### Effect on Pasting Property of Corn Starch

The pasting property of corn starch (CS), the mixture of CS with different concentrations of CIDF (5, 10, 20, 30, 40, and 50%), and a mixture of IDF/CS, MIDF/CS, and CIDF/CS were determined by a rapid viscosity analyzer (RVA) (Newport Scientific, NSW, Australia). The test procedure of the RVA assay was performed according to a previous study (30). The mixture was dispersed in 25 ml distilled water, and the temperature program was as follows: the slurry was held at 50°C for 1 min, and then, the temperature was increased to 95°C and held for 2.5 min and finally cooled down to 50°C and held for 2 min. The mixture was stirred by a plastic paddle at 960 rpm in the first 10 s and revolved at 160 rpm during the rest of the process. During the pasting, the values of peak viscosity (PV), breakdown viscosity (BV), final viscosity (FV), setback viscosity (SV), peak time (PT), and pasting temperature (PaT) were recorded by RVA.

### Glucose Diffusion Inhibition

Based on the reported method (7) with some modifications, 0.2 g of sample was mixed with 10 ml of 100 mmol/L glucose solution and placed the mixture into a 10-cm dialysis bag (cutoff molecular weight of 8,000) with 200 ml of distilled water as the dialysis solution. The dialysis system was incubated at 37°C. A sample of 500 μl dialysis solution was taken at 10, 20, 30, 60, 90, 120, 150, and 180 min. The glucose content of the dialysis solution was determined with a biosensor analyzer. The control group was treated without a sample.

### Effect on *in vitro* Starch Digestion

The effect of IDF, MIDF, and CIDF on starch digestion was determined based on the previous method (31); 50 mg of CS and 20 mg of sample were mixed with 10 ml of HAc-NaAc (0.5 M pH 5.2) and incubated at 37°C for 10 min until thoroughly mixed. Then, 4 ml α-amylase and 1 ml glucosidase were added and incubated at 37°C. The solution was collected and then mixed with 500 μl of Na_2_CO_3_ to inactive the enzyme after incubation for 10, 20, 30, and 60 min. The solution was centrifuged at 4,000 rpm for 10 min, and the glucose content of the supernatant was then measured using a biosensor analyzer to estimate the effect of the IDF sample on starch digestion. The control group was carried out without the addition of a sample.

### α-Amylase Activity Inhibition Capacity

According to the previous method with some modifications (32), 20 mg IDF and 50 mg CS were mixed with 10 ml HAc-NaAc (0.5 M pH 5.2), and then, 4 ml α-amylase was added and incubated the mixture with shaking in a water bath for 1 h, followed by boiling at hot water to inactive the enzyme. Then, the solution was centrifuged at 4,000 rpm for 10 min to collect the supernatant and determine the glucose content with a biosensor analyzer. The control group was carried out without the addition of a sample.

### Statistical Analysis

A one-way fixed-effects analysis of variance (ANOVA) test was performed using statistical software (SPSS version 18.0, SPSS Inc., Chicago, IL, USA). All trials were done in triplicate, and the statistical means and standard deviations were calculated and shown.

## Results and Discussion

### Preparation of IDF, MIDF, and CIDF

**Figure 1:**
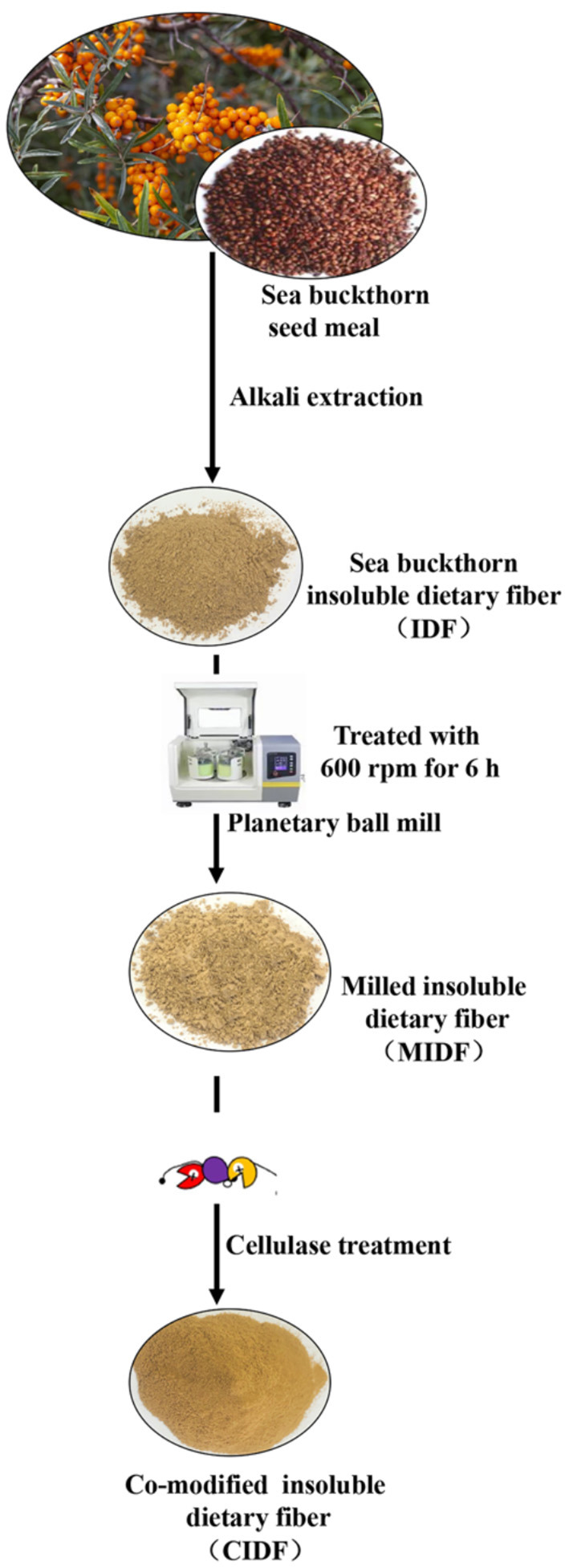
The flowchart of the preparation of MIDF and CIDF.

A schematic showing the preparation method of IDFs is shown in Figure 1. IDF was extracted from sea buckthorn seed meal through alkali extraction, which is an effective way to release the soluble components, such as polysaccharides and pectin (33). The IDF was extracted by the alkali method at a yield of 51.3%. Compared with crude grinding, ball milling powder exhibits smaller particles, larger surfaces, better dispersibility, and higher bioavailability (27). It could also destroy the connection among lignin, cellulose, and hemicellulose and reduce the amount of lignocellulose to increase the accessibility of cellulose to enzymes (19). As shown in Table 1, a planetary mill was used to get the MIDF at the particle size of 85.33 ± 5.5 μm, which decreased by 33.1% compared to IDF (104.6 ± 6.4 μm). Enzymatic hydrolysis is a usual means to modify IDF to get a sparse structure and expose relevant functional groups, such as hydroxyl and carboxyl groups (16). Thus, cellulase was used to treat MIDF to get the CIDF.

**Table 1:** Particle sizes of IDF and MIDF, the physicochemical properties (WHC, OBC, SW, BD, and angle of repose) of IDF, MIDF, and CIDF.

	**IDF**	**MIDF**	**CIDF**
D10 (μm)	6.4 ± 0.1^a^	4.1 ± 0.1^b^	
D50 (μm)	122.1 ± 6.3^a^	58.2 ± 10.9^b^	
D90 (μm)	198.1 ± 19.4^a^	162.4 ± 36.2^b^	
Mean (μm)	104.5 ± 6.6^a^	72.0 ± 14.9^b^	
Specific surface area (μm^2^/μm^3^)	0.3 ± 0.0^b^	0.5 ± 0.0^a^	
WHC	2.6 ± 0.1^b^	3.1 ± 0.1^b^	3.6 ± 0.4^a^
OBC	1.8 ± 0.2^b^	2.1 ± 0.1^ab^	2.2 ± 0.1^a^
SC	1.1 ± 0.1^b^	1.3 ± 0.2^ab^	1.6 ± 0.1^a^
Bulk density (g/ml)	0.42 ± 0.0^b^	0.56 ± 0.1^a^	0.39 ± 0.1^c^
Angle of repose (°)	42.4 ± 2.9^b^	46.5 ± 1.9^a^	29.1 ± 1.3^c^

*Different letters in the same row represent significant differences at p < 0.05*.

### Structure Characterization

The microstructure of IDF is known to change depending on the modifications. This also influences the physicochemical properties and special functional groups including -OH and -COOH (34). Consequently, the effects of ball milling and combined treatment on IDF could be concluded from the microstructure changes.

#### Scanning Electron Microscopy

**Figure 2:**
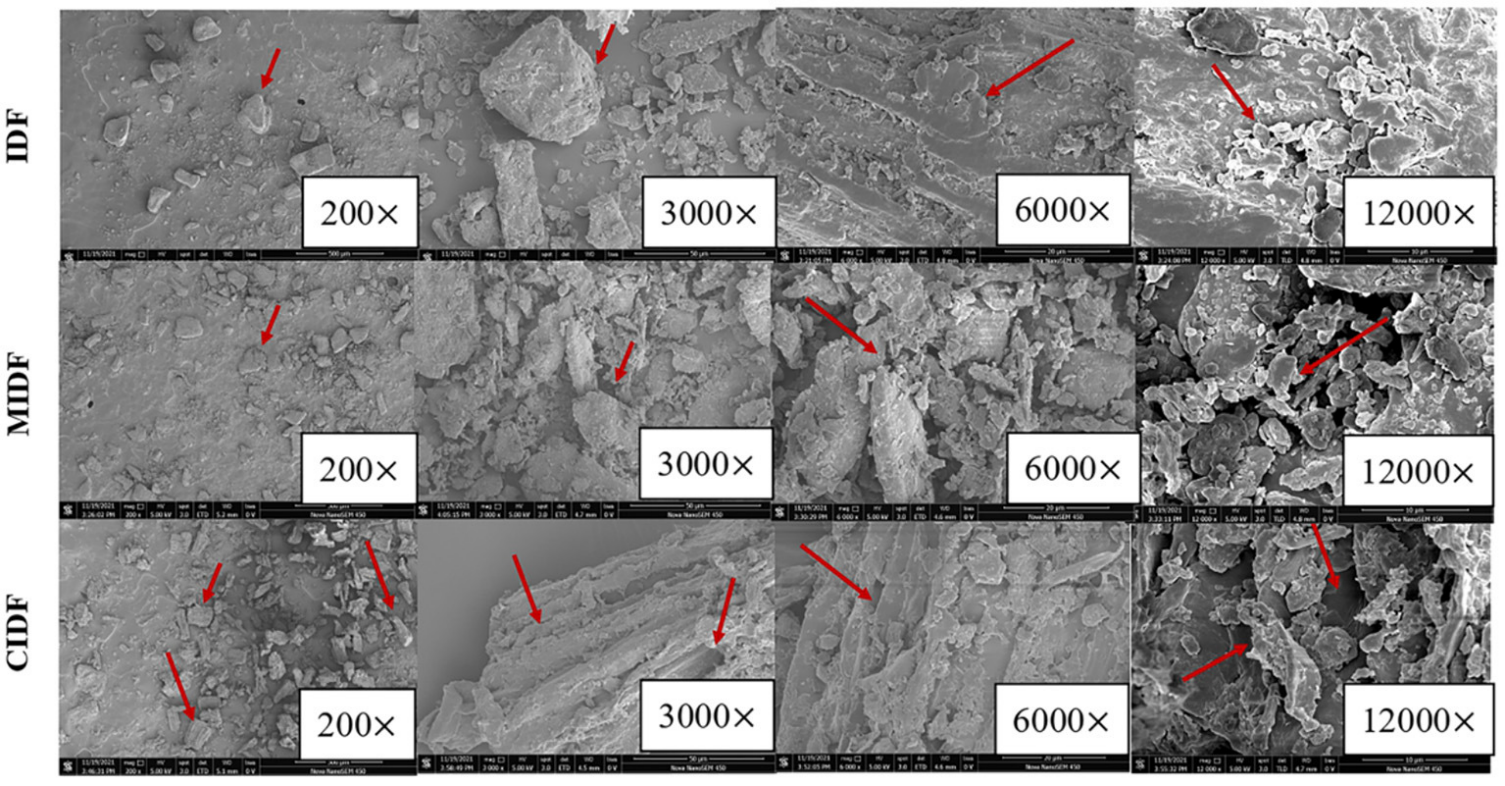
SEM of insoluble dietary fibers with the magnification of 200×, 3,000×, 6,000×, and 12,000×.

Scanning electron microscopy images of IDF, MIDF, and CIDF were taken and shown in Figure 2. IDF showed a larger particle size and had a smoother and more compact surface. Due to the strong external forces during the ball milling process, larger fiber particles were broken into smaller particles, which not only caused greater changes to the structure of the fiber but also had a greater impact on the functional properties of the IDF (27). MIDF had smaller particles and a looser structure compared with IDF. Cellulase treatment could break the glycosidic bonds, degrade the polysaccharides in the cell wall, and improve the property of IDF (34). In this study, CIDF obtained a wrinkled surface, loose and sparse porous structure according to Figure 2. Many tiny fragments could also be observed on the surface of MIDF and CIDF, suggesting that violent tearing occurred during modifications. Cellulase combined with ball milling brought destruction to the wall polysaccharide chains, thus increasing the surface area of modified IDFs. Therefore, modifications in this study significantly changed the microstructure of IDFs. Changes in microstructure could also influence the hydration properties of IDFs (35).

#### FT-IR

**Figure 3:**
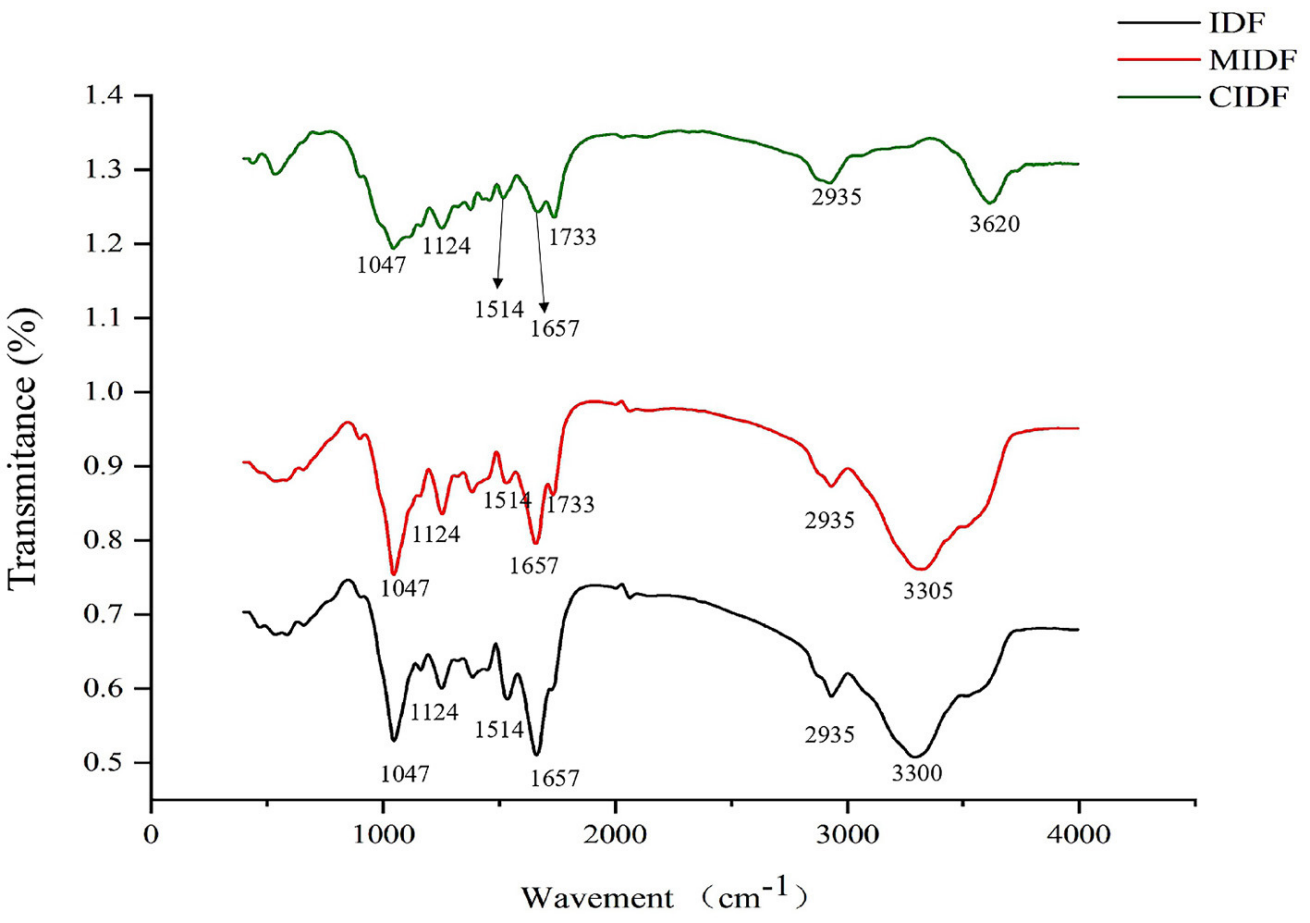
FT-IR spectra of IDFs.

From the infrared spectrum of IDF, MIDF, and CIDF, in Figure 3, the structural analysis could be concluded. All of the samples presented similar spectra and special characteristic bands in absorption peaks. For all fibers, the wind bands around 3,500 cm^−1^ were mainly caused by O-H in hydroxyl groups. It was apparent that the modifications enhanced it, which might be related to the increase in specific surface and exposed hydrogen bonds (36). The band at 2,935 cm^−1^ represented the C-H in samples, and absorption peaks at 1,514 cm^−1^ were related to the stretching and vibration of carboxyl groups (37). Moreover, the bands at 1,657 and 1,733 cm^−1^ corresponded to C=O in IDFs. Additionally, C-O-C in cellulose and lignin were observed at 1,047 and 1,124 cm^−1^ (38), and the intensities of those bands decreased with the modifications, indicating that the treatment broke down the intermolecular forces in the fibers and disintegrated the IDF structure. Absorption peaks from 500 to 1,000 cm^−1^ were due to the vibration of carbohydrates in IDFs (18). In summary, modifications in this study did not change the type of functional groups in IDFs, whereas the treatments provided MIDF and CIDF with different intensities of several functional groups, such as increasing the amount of hydroxyl and carboxyl groups.

#### XRD

**Figure 4:**
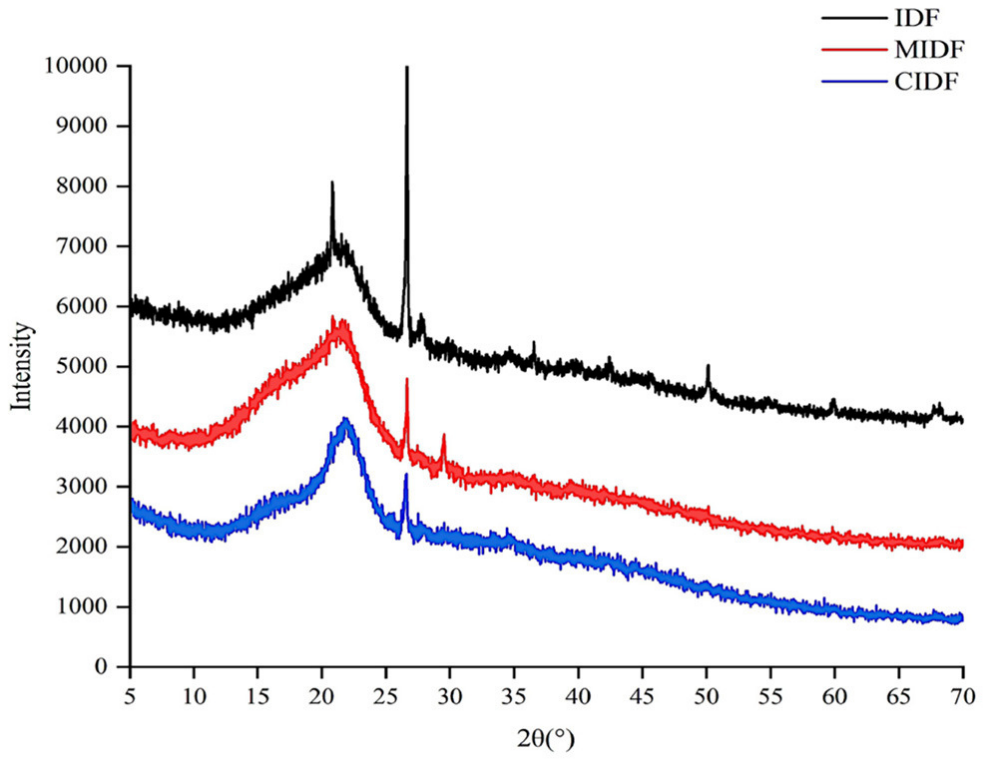
XRD patterns of insoluble dietary fibers.

X-ray diffraction could be used to determine the crystal structure of IDFs, with different crystal structures corresponding to different diffraction angles (39). Therefore, in this study, XRD was used to examine the aggregation state of the IDFs and to characterize the changes in relative crystallinity of fibers after the modifications. Results in Figure 4 exhibited the typical cellulose form in three samples with relative peaks of different intensities. It is reported that IDF is mainly composed of a crystalline region due to the existence of cellulose and a non-crystalline region due to the composition of hemicellulose and lignin (40). The strong peak around 21 and 26° in Figure 4 was the main diffraction peak indicating the typical cellulose crystal in insoluble dietary fiber. The peaks decreased with the modification, indicating ball milling and the ball milling combined with cellulase treatment decreased the amorphous area of the IDFs. Additionally, several small peaks from 30 to 70° existed in IDF but disappeared in MIDF and CIDF, which suggested the degradation of the crystallization zone. A previous study had reported that after degradation, the crystal structure becomes disordered and the intermolecular interaction decreased (41). In summary, MIDF and CIDF were looser in structure and weaker at the intermolecular level, which might lead to the improvement in hydrophilic and lipophilic ability, adsorption capacity and explaining the better property of the modified samples.

### Physicochemical Properties

#### Modification Effects on the SC, WHC, and OBC of the Fibers

The ability to retain oil and water is essential for the application of IDF, and this property is related to its structure (42). In this study, with the modification of IDF, relevant properties enhanced a lot. According to the particle size of IDF and MIDF in Figure 1, it was obvious that ball milling was an effective method to grind IDF into smaller particles. The mean particle size of MIDF was 78.6 ± 5.5 μm and decreased by 24.4% compared to the size of IDF at the mean particle size at 104.6 ± 6.4 μm. The specific surface of MIDF also increased by 62.5% compared to IDF. The WHC, OBC, and SC of IDF, MIDF, and CIDF are also shown in Table 1. Compared to IDF, the WHC, OBC, and SC of MIDF were enhanced by 16.13, 14.29, and 15.38%, respectively. WHC, OBC, and SC for CIDF improved by 38.5, 22.2, and 25.0%, respectively, compared to IDF. Modification with ball milling combined with cellulase treatment could improve the physicochemical properties of IDF, which might be due to the change in structure and expose more hydrophilic groups according to the structural analysis. The increase in WHC and SC might be due to the treatments disrupting the surface structure of the IDF, allowing the internal hydrophilic groups, such as hydroxyl groups, to be fully exposed, and thus leading to a change in the hydration capacity (43). Besides, the slight improvement in OBC after treatments could be concluded from Table 1. This suggested that modifications could also break down the interactions inside fibers, thus making the structure looser and letting oil seep into the hydrophobic zone (44). Combined with the microstructural analysis, the wrinkled surface of CIDF provided it with a looser structure, which improved the surface characteristics, resulting to the higher WHC, OBC, and SC. The properties of MIDF have also been improved due to the smaller particle size and higher specific surface. The previous study about the physical and enzyme treatment on IDF of wheat bran also obtained better WHC, OBC, and SC, which demonstrated that enzyme modification could be effective in improving the physicochemical properties of IDF (14).

#### Modification Effects on the BD, Angle of Repose of the Fibers

From the Table 1, the BD of IDF, MIDF, and CIDF were 0.42 ± 0.0, 0.56 ± 0.1, and 0.39 ± 0.1 g/ml, respectively. The more uniform the size and shape distribution of IDF, the smaller the gap between particles when stacking. Besides, with the specific surface area, surface aggregation force, and mutual gravitational force between IDF particles increased, particles became easier to adsorb and agglomerate with each other (27). The increase of BD of modified IDFs may be related to the enhanced interaction between the sample particles, which was consistent with the results presented in the structural analysis. As for CIDF, cellulase treatment provided it with a porous structure, the disintegration of the amorphous zone made it less dense and the grooved surface allowed for increased permeability among the particles, leading to the decrease of BD (45). The angle of repose of powder is an indicator of the variation in the powder's fluidity, the greater the angle of repose, the worse the flowability of the powder (46). The results in Table 1 showed that the repose angle of IDF, MIDF, and CIDF was 42.4, 46.5, and 29.1°. The results here showed that the ball milling made the fluidity of MIDF worse. The reason for the poor flowability was that as the particle size decreased, the specific surface area of the particles increased, the surface polymerization force increased, and the electrostatic attraction between the powder molecules increased (46). Whereas with the treatment of ball milling combined with cellulase, the surface of CIDF appeared winkled and the structure got looser, the permeability increased and compressibility decreased, which resulted in the improvement of flowability (28). A previous study about modified soybean residue IDF also found that enzyme treatment could reduce its BD and porosity (44).

#### Cation Exchange Capacity

**Figure 5:**
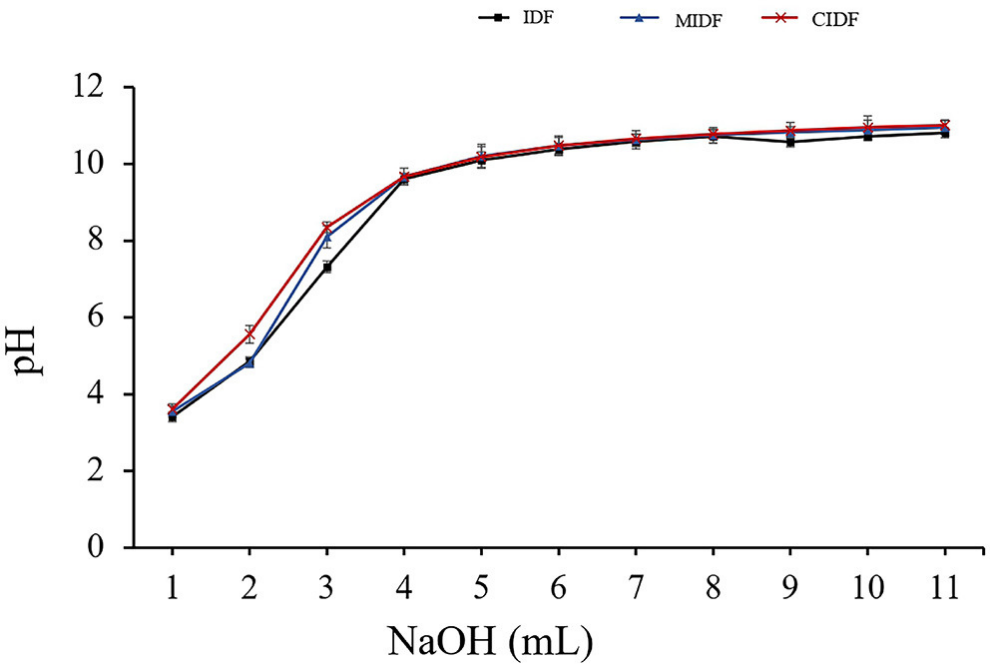
Cation exchange capacity of insoluble dietary fibers.

Insoluble dietary fiber contains cellulose, hemicellulose, and lignin, which have numerous carboxyl and hydroxyl groups. These functional groups tend to produce the capacity of cation exchange, which could lower blood pressure and bind heavy metal ions, thus protecting the body (47). As shown in Figure 5, the IDFs' pH tended to increase with the addition of NaOH. The pH of MIDF (3.5–10.9) and CIDF (3.6–11.1) solution both increased quickly with the NaOH addition and kept increasing for a longer time than IDF (3.4–10.8). According to the structural analysis, ball milling endowed MIDF with smaller particle size and higher specific surface area, exposing more carboxyl and hydroxyl groups, which improved the cation exchange capacity. Cellulase treatment of IDF is effective in enlarging the pores in the fiber cell wall and exposing more polar groups (48). The comodifications provided a special structure for CIDF with the increased number of -OH and -COOH groups and decreased steric effects, leading to the higher cation adsorption ability and faster changes with the addition of NaOH. The previous study about insoluble dietary fibers of ginger residue modified by cellulase also exhibited a great ability in cation exchanging (18). This showed that the enzymatic treatment was a powerful way to improve the cation exchange capacity of IDFs.

### *In vitro* Hypoglycemic Properties

#### Effects on Pasting Properties

**Figure 6:**
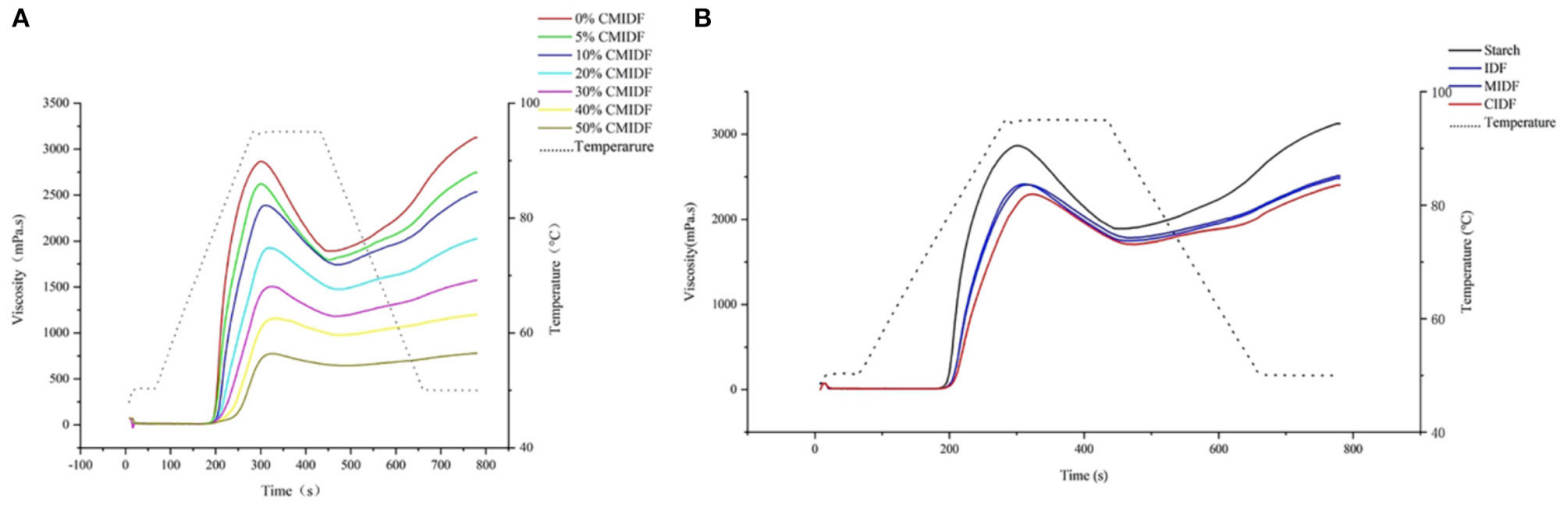
Pasting curves of replacement of CS with insoluble dietary fibers. **(A)** Effect of different addition of CIDF. **(B)** Effect of addition with IDF, MIDF, and CIDF.

Starch is one of the most widely used ingredients in food, and the addition of IDF affects its physicochemical properties, such as water retention, thermodynamic properties, rheology and texture, and in particular the pasting property (30). The study of IDF addition on the pasting property of starch is important for its implications in starch-based food processing and quality control. To investigate the effect of insoluble dietary fiber on the starch pasting property, CS was mixed with 0, 5, 10, 20, 30, 40, and 50% CIDF, and the results are shown in Figure 6A. CS had a high viscosity during pasting, whereas with the addition of CIDF, the total viscosity decreased. The PV is related to the swelling of the particles during pasting (49). The PV decreased with the addition of more CIDF because the increase in fiber content reduced the starch content, thus making less starch available for pasting. IDF has a certain WHC, thus making less water available in the system and making starch pasting more difficult. The BV is related to the ability to resist shear and high temperature (50). With the addition of CIDF, the BV decreased significantly. This indicated that the addition of CIDF could improve the thermal stability of starch. SV is calculated by the viscosity of the starch solution during cooling, which indicates the aging of the starch (51). Besides, the viscosity varied depending on the fiber content. When fiber content was <10%, the viscosity of the fiber partially compensated for the change in the viscosity of starch and the viscosity changed insignificantly (*p* > 0.05). When the addition exceeded 10%, the viscosity of the fiber was not sufficient to compensate for the reduction in starch pasting and the viscosity decreased significantly (*p* < 0.05).

Subsequently, IDF, MIDF, and CIDF were added at 10% of starch mass to evaluate the effects of different fibers on the starch pasting properties. The results are shown in Figure 6B. MIDF and IDF had less effect on the pasting of starch, and the pasting process was similar, indicating that the reduction in particle size within this range did not have a significant additional effect on starch pasting (47). The mixture with the addition of CIDF showed a significant viscosity decrease during the pasting process compared to IDF. This may be due to the looser structure of CIDF and the exposure of more -OH and -COOH groups on the surface, which provided it with stronger WHC and SC. As a result, CIDF could absorb water from the system more efficiently and limit the solubility of amylose. Furthermore, compared to the control group, the PV of CIDF/CS mixture decreased by 17.7%, and the BV and SV decreased by 39.6 and 37.1%, respectively. This indicated that a higher thermal stability of the mixture and the pasting and aging of starch were more difficult (52). The previous study on the effect of fiber-rich apple pomace on starch pasting also showed that the addition of insoluble particles could significantly change the pasting properties of a starch-based system (30).

#### Glucose Adsorption Capacity

**Figure 7:**
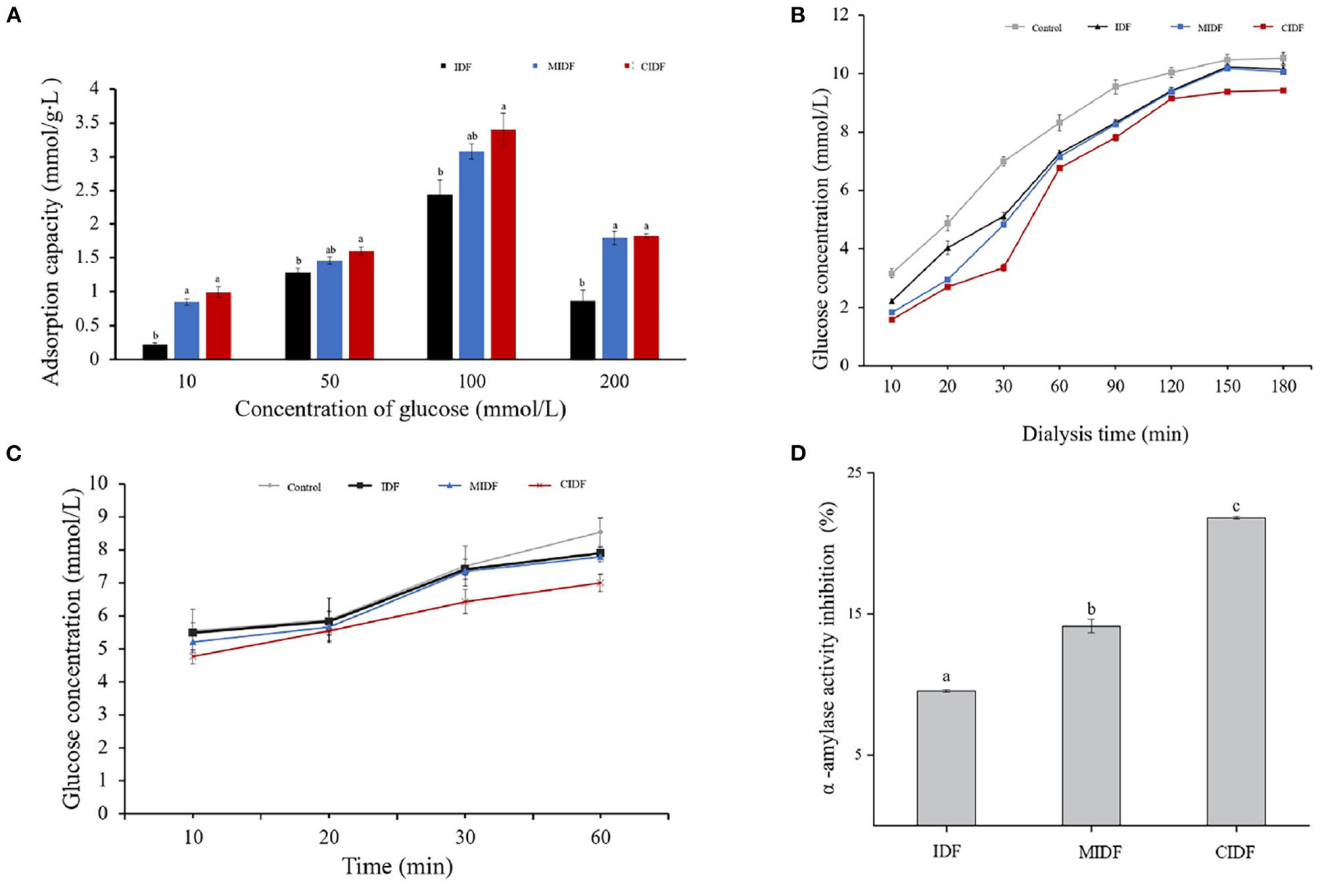
*In vitro* hypoglycemic properties of IDFs. **(A)** Glucose adsorption capacity of IDF, MIDF, and CIDF. **(B)** Glucose diffusion inhibition of IDF, MIDF, and CIDF. **(C)** Effect on starch digestion of IDF, MIDF, and CIDF. **(D)** α-amylase activity inhibition of IDF, MIDF, and CIDF.

The previous study has reported that IDF from wheat bran with modification by several methods also had great capacity in absorbing glucose in solution due to the changes in structure (14). Different concentrations of glucose solution also resulted in different adsorption capacities of IDF. The glucose adsorption capacities are shown in Figure 7A. It was obvious that modified IDFs had a better ability in absorbing glucose. With the increase of glucose concentrations (10–200 mmol/L), the glucose absorbed by IDF, MIDF, and CIDF was 0.2–2.5, 0.3–3.1, and 0.4–3.4 mmol/g•L, respectively. The best adsorption capacity of IDFs was achieved when the glucose solution concentration was 100 mmol/L. Compared to IDF, the glucose adsorption of MIDF and CIDF increased by 24 and 36%, respectively. From the structural analysis, ball milling treatment decreased the particle size of MIDF, the specific surface area increased and exposed more functional groups, such as hydroxyl groups, and thus, the Van der Waals and hydrogen-bonding forces between MIDF and glucose molecules were enhanced (53). As for CIDF, in addition to the enhanced interaction between CIDF and glucose molecules, the porous structure allowed CIDF particles to form a network structure. This made it easier for glucose molecules to enter the interior of CIDF and therefore adsorbed more glucose (14). Besides, Figure 7A also shows that in different concentrations of glucose solution, the CIDF exhibited different adsorption capacities. The results implied that IDFs could still absorb a small amount of glucose even in a low concentration. MIDF and CIDF were beneficial in reducing the amount of available glucose in the intestinal lumen, and modification by milling combined with cellulase treatment was an effective way to enhance the adsorption property of dietary fiber extracted from sea buckthorn seed meal.

#### Glucose Diffusion Inhibition

The glucose diffusion test simulates the diffusion and absorption of glucose in the human intestine and evaluates the effect of the samples on reducing postprandial blood glucose level *in vitro* (7). It was reported that IDFs were beneficial in inhibiting glucose diffusion. The mechanism of glucose diffusion inhibition by dietary fiber could be divided into two main aspects: (1) soluble dietary fiber with viscous properties increases the viscosity of the system, thereby inhibiting the diffusion of glucose; (2) IDF has a physical adsorption effect on glucose, causing glucose to be adsorbed into the pores of the fiber and thereby reducing the diffusion rate of glucose (54). Also, the barrier effect of the particles of the IDF on the glucose and the crosslinked structure in the fiber have a binding effect on glucose molecules (55). In this study, the glucose concentration of dialysate was determined to evaluate the glucose dialysis retardation capacity of the samples, and the results are shown in Figure 7B. As time increased from 10 to 180 min, the glucose in dialysate without any sample was enhancing constantly. In contrast, the addition of the IDFs reduced the amount of glucose diffusion in the dialysate during the 180-min dialysis test compared to the control group. It was obvious that with CIDF addition (1.6–9.4 mmol/L), the glucose concentration was lower than the other three groups. The glucose concentrations of IDF (2.2–10.2 mmol/L) and MIDF (1.8–10.1 mmol/L) groups showed only a slight decrease related to the control (3.2–10.5 mmol/L). This indicated that all three samples had the binding capacity for glucose, and CIDF obtained from the combined modification treatment had the best and fastest binding effect. Combined with the structural analysis, the reason for this may be that the wrinkled surface of CIDF exposed more -OH and -COOH groups which could effectively adsorb glucose molecules (55). Besides, the barrier effects of CIDF particles also delayed the diffusion of glucose (7). A previous study has reported that IDF from coconut cake with modification also showed excellent glucose diffusion inhibition capacity due to the special structure, indicating that combined modification with enzyme and physical treatment was essential to improve the property of IDF (7).

#### Effect on Starch Digestion

In this study, the effects of IDF, MIDF, and CIDF on starch digestion were demonstrated according to the changes in glucose contents as digestion time increased. As shown in Figure 7C, as the time increased, changes in glucose contents in the different groups were obvious. It could be concluded that with IDF and MIDF addition, there was no significant difference compared to the control group from 10 to 30 min. Whereas digestion time increased to 60 min, it was obvious that all three samples could inhibit the digestion continuously. As for CIDF, it exhibited a better inhibition capacity during the whole digestion, which demonstrated that cellulase treatment combined with ball milling could improve the effect of CIDF on starch digestion. Summaries could be concluded that both IDFs in this study had effects on inhibiting starch digestion but CIDF showed a better inhibition. The inhibition mechanism might be due to the enzyme inhibitors on the IDFs' surface acting directly on the relevant enzymes thus inactive them (56). According to the structural analysis, the structure of CIDF was sparse and the gaps among the particles were increased due to the combined modification and therefore has a stronger WHC, which made the system thicker during digestion and inhibited the movement of amylase. In addition, the wrinkled surface of CIDF exposed more binding sites to enzymes, thereby blocking the activity of enzymes and inhibiting the contact of enzymes with starch (3). The decrease of particle size in MIDF was not significant in the inhibition of starch digestion compared to IDF (32). It has been reported that modified dietary fiber from *Nannochloropsis oceanica* could inhibit starch digestion by inhibiting the activity of amylase (29). Further investigation about the inhibition mechanism on enzymes is needed.

#### α-Amylase Activity Inhibition

α-amylase is an important factor in the digestive conversion of starch to glucose in the intestine (3). IDF was considered to be a good α-amylase inhibitor extracted from natural materials due to its unique structure and properties according to the previous study (56). From Figure 7D, the inhibitory activity was demonstrated by the glucose content. It could be concluded that all three IDFs exhibited inhibition on α-amylase activity. Besides, CIDF showed the strongest inhibitory activity at 21.8% compared to IDF (9.5%) and MIDF (14.1%). This phenomenon was consistent with the results of the effect of IDFs on starch digestion, suggesting that the inhibition of starch digestion by samples in this study was mainly derived from the inhibition of α-amylase. Moreover, results also indicated that ball milling treatment and ball milling combined with cellulase treatment significantly increased the α-amylase activity inhibition of IDF. In conjunction with structural analysis, such results may be due to the looser structure and increased porosity of the modified IDFs, thus increasing the adsorption capacity for α-amylase. In addition, CIDF also obtained better WHC and SC, which could absorb water and swell sufficiently to form a barrier and reduce the contact between starch and α-amylase. The wrinkled surface of CIDF also exposed more polar groups (such as hydroxyl and carboxyl groups) and the active sites of α-amylase, and thus, CIDF could bind to a portion of α-amylase and inhibit its activity (56). The previous study has also reported that IDF of rice bran could inhibit the activity of α-amylase by adsorbing it and preventing its contact with starch. Besides, the surface of rice bran IDF contained enzyme inhibitors, which could act directly on α-amylase and reduce its activity (3). The results in this investigation suggested that CIDF might influence digestion by inhibiting the α-amylase action with the starch.

## Conclusion

Modifications were applied to sea buckthorn IDF to enhance its *in vitro* hypoglycemic capacity. IDF, MIDF, and CIDF were obtained by alkali extraction, modification with ball milling, and ball milling combined with cellulase treatment. It has been proven that the modifications used in this study could significantly increase the glucose adsorption capacity and have an influence on starch digestion, thus having an *in vitro* hypoglycemic effect. FT-IR, SEM, and XRD demonstrated that the treatments could change the microstructure of IDF and obtain a looser structure, which was positively correlated with glucose absorption during starch digestion in the intestine. Compared with the IDF, modified IDF exhibited superior performance in many aspects. Therefore, ball milling and ball milling combined with cellulase treatment could improve the properties of IDF and provide a basis for the in-depth exploitation of sea buckthorn resources. Considerably, much more work will need to be done to determine the *in vivo* function of modified IDF in the future.

## Data Availability Statement

The original contributions presented in the study are included in the article/supplementary material, further inquiries can be directed to the corresponding author/s.

## Author Contributions

YZ performed methodology, validation, formal analysis, investigation, data curation, and writing original draft preparation. XJ was involved in visualization and funding acquisition. MW did the conceptualization and funding acquisition. DS contributed in writing, reviewing, and editing. QP was involved in conceptualization and project administration. All authors contributed to the article and approved the submitted version.

## Funding

This research was financially supported by the Henan Key Laboratory of Cold Chain Food Quality and Safety Control (CCFQ 2021), Beijing Engineering and Technology Research Center of Food Additives, Beijing Technology and Business University (BTBU), Yulin City Science and Technology Plan Project (No. CXY-2020-074), and Science and Technology Planning Project of Xining (No. 2021-Y-15).

## Conflict of Interest

MY, TY, and HY were employed by Puredia Limited. The remaining authors declare that the research was conducted in the absence of any commercial or financial relationships that could be construed as a potential conflict of interest.

## Publisher's Note

All claims expressed in this article are solely those of the authors and do not necessarily represent those of their affiliated organizations, or those of the publisher, the editors and the reviewers. Any product that may be evaluated in this article, or claim that may be made by its manufacturer, is not guaranteed or endorsed by the publisher.
